# Assessment of the efficacy of SERI osteotomy for hallux valgus correction

**DOI:** 10.1186/s13018-019-1067-3

**Published:** 2019-01-24

**Authors:** Talal Almalki, Raheef Alatassi, Ahmad Alajlan, Khalid Alghamdi, Abdullah Abdulaal

**Affiliations:** 1 Department of Orthopedic Surgery, Security Forces Hospital, P.O. Box: 3643, Riyadh, 11481 Saudi Arabia; 2 College of Medicine, King Saud bin Abdulaziz University for Health Sciences,, Riyadh, Saudi Arabia

**Keywords:** SERI, Hallux valgus, Correction surgery, Radiography, Outcomes, Complications, Congruency, Sesamoid

## Abstract

**Background:**

SERI (*S*imple, *E*ffective, *R*apid, and *I*nexpensive) osteotomy is an accepted minimally invasive distal first metatarsal osteotomy performed to correct hallux valgus (HV). In the absence of reports of efficacy of the SERI technique in the Middle East, we studied 1-year outcomes of SERI osteotomy performed at our hospital in Saudi Arabia.

**Methods:**

We reviewed the medical charts of patients aged 20 to 60 years who underwent SERI osteotomy for HV between August 2013 and September 2016 and identified 29 patients, 2 (6.9%) men and 27 (93.1%) women, who met the criteria for inclusion in the study. Patients’ clinical and operative characteristics were examined, their pre- and postoperative (1-year) radiographic measurements were compared, and the occurrence of any postoperative complication/event was noted.

**Result:**

Patients’ mean age was 34.9 ± 13.6 years. Six patients (20.7%) were treated for severe HV. Mean operation time was 11.1 ± 2.3 min. Four patients (13.8%) reported postoperative pain. No revision surgery was done. Congruency of the hallux metatarsophalangeal joint increased significantly, documented in only 4 patients (13.8%) preoperatively but in 17 (58.6%) at 1 year. The mean hallux valgus angle (HVA), intermetatarsal angle (IMA), and distal metatarsal articular *angle* (DMAA) were significantly decreased at 1 year. The HVA normalized in 20 patients (69.0%), the IMA normalized in 25 patients (86.2%), but the DMAA normalized in only 4 patients (13.8%). The number of patients with sesamoid subluxation decreased from 29 (100%) to 13 (44.8%).

**Conclusion:**

Our study data indicate that SERI osteotomy reliably reduces a wide spectrum of HV deformities and it is a safe procedure with very minimal complications.

**Trial registration:**

This study is registered in ClinicalTrials.gov under the following reference number: NCT03669900.

## Introduction

Hallux valgus (HV) is a complex deformity that involves lateral deviation of the proximal phalanx on the first metatarsal head, is frequently associated with medial deviation of the first metatarsal, and can be accompanied by significant functional disability and foot pain [1]. Reported estimates of the global prevalence are 23% among adults ≤ 65 years of age and 35.7% among persons > 65 years of age, higher among females than among males, and directly proportional to increasing age [2]. The reported estimate in the USA stands at 28.4% [3].

Surgery is indicated for HV when functional disability or severe or persistent foot pain that interferes with activities of daily living is present and conservative treatment has failed. The degree of deformity, based on the results of radiographic assessment and physical examination, is also taken into consideration [1]. Radiographic assessment includes evaluation of weight-bearing anteroposterior (AP) and lateral images of the foot. The severity of the deformity is usually classified as mild when the hallux valgus angle (HVA) is 15 to 19° and intermetatarsal angle (IMA) is 9 to 13°, moderate when the HVA is 20 to 40°, and severe when the HVA is > 40° and the IMA is > 20° [4]. Presently, there are several number of operative procedures recognized for treatment of HV, including the Keller procedure, the distal soft-tissue procedure, various osteotomies of the first metatarsal, various distal metatarsal osteotomies (the Wilson procedure, Mitchell osteotomy, distal chevron osteotomy), diaphyseal osteotomy performed with arthrodesis [4], and many others. Most of these surgical procedures have been shown to provide morphologic and functional rebalance. There are more than 150 surgical procedures described to treat HV, but none of them is considered the gold standard, and each has its own advantages and disadvantages [5]. Minimally invasive techniques used for correction of HV include arthroscopy, percutaneous osteotomy, and minimum incision osteotomy, and these have been found to improve outcomes by decreasing recovery and rehabilitation times [6].

SERI (*S*imple, *E*ffective, *R*apid, and *I*nexpensive) osteotomy is a minimally invasive surgery that has been known as Bosch osteotomy and shown by several authors to possess the advantages of percutaneous techniques but to involve less tissue dissection and to require only temporary hardware. In addition, no particular instrumentation is needed, and the surgery is performed under direct vision without fluoroscopy [7–11]. It is a type of distal first metatarsal osteotomy that often includes lateral soft tissue manipulation to position the sesamoids and release the lateral structures [12]. Several studies of SERI have shown good correction of the deformity with very few complications, such as avascular necrosis of the metatarsal head or pseudoarthrosis, and the recurrence rate has been low [7, 10, 11, 13]. The SERI technique has been shown to be effective in treating mild to moderate HV, and the reported cost-effectiveness ratio is relatively low [9, 14–16]. Patients’ American Orthopedic Foot and Ankle Surgery (AOFAS) scores have increased from a mean preoperative value of 43 to a postoperative follow-up value of 88, and the mean HVA, IMA, and distal metatarsal articular angle (DMAA) have decreased from the preoperative measures of 33°, 13°, and 20°, respectively, to 16°, 7°, and 8°, respectively [12, 13]. Also reported is achievement of mean HVA correction to 11.8° and of mean IMA correction to 6.3°, as measured on weight-bearing AP foot radiographs [17]. Whereas an HVA of < 15° is normal, 15 to 19° is regarded as mild deformity, 20 to 40° as moderate deformity, and > 40° as severe deformity. An IMA of < 9° is normal, whereas 9 to 11° is regarded as mild deformity, 11 to 16^o^ as moderate deformity, and > 16° as severe deformity; a DMAA of ≤ 8° is normal [18]. Studies have shown significant improvements in 4 of 5 American Academy of Orthopedic Surgeons and AOFAS scores, like those seen following other surgeries [19]. Furthermore, the SERI technique, in comparison to other procedures, provides for correction of the HVA, IMA, and DMAA with a smaller skin incision, less operation time, less expensive fixation device(s), and absence of residual pain attributable to the hardware [5]. The validity of the AOFAS score in assessing the hallux metatarsophalangeal (MTP) joint and lesser toe MTP joints is, however, controversial [20].

Radiographic assessment of specific variables, including the HV angles, position of the sesamoids, and articular congruency, has been shown to ensure adequate correction of angular HV deformities [21]. Preoperative measurement of the sesamoid positions on the axial view radiograph can guide surgeons in choosing the appropriate surgical technique [22]. Furthermore, measuring the HVA and the IMA has been recommended for both preoperative assessment of the severity of HV and postoperative outcome assessment [23]. To the best of our knowledge, there is no report of application of the SERI technique for treatment of HV in Saudi Arabia or any part of the Middle East. Therefore, we conducted a study in which we compared radiographic measurements obtained preoperatively and at 1 year postoperatively, evaluated complications, and determined the cost-effectiveness of SERI osteotomy performed at our hospital over a 3-year period.

## Methods

We reviewed the medical charts of all patients with HV who were treated by SERI osteotomy during the period August 2013 through September 2016 in the Department of Orthopedic Surgery at the Security Forces Hospital in Riyadh, Saudi Arabia. We identified 29 patients, 2 (6.9%) men and 27 (93.1%) women, for inclusion in the study. These were patients who met the following study criteria: age ranging from 20 to 60 years, a diagnosis of mild or moderate HV judged to be reducible, an HVA of ≤ 40° and IMA of ≤ 20°, arthritis of the first MTP joint up to Regnauld Grade 2, and postsurgical follow-up of at least 1 year at the Security Forces Hospital. Patients who presented with stiffness of the first MTP joint, severe arthritis of the first MTP joint (>Regnauld Grade 2), or a history of rheumatoid arthritis or another inflammatory disease, diabetes, or a neurological disorder, and patients who had undergone a previous hallux surgery were excluded from the study.

### Operative technique

The surgery consisted of varus traction, skin incision, metatarsal osteotomy, and K-wire insertion. All surgeries were performed by the senior consultant orthopedic surgeon, as were the preoperative planning and the clinical follow-up. This surgeon was assisted by at least one other orthopedic surgeon.

For the surgery, the patient was placed in the supine position, and a tourniquet was applied to the patient’s leg. Spinal or regional anesthesia was induced. The procedure began with application of a varus force for 2 to 3 min on the first MTP joint to stretch the lateral capsule, and this was followed by placement of a 2-cm medial incision just proximal to the medial eminence at the level of the neck of the first metatarsal bone. The capsule was then opened to expose the neck of the metatarsal bone. Two small retractors were used to fully expose the osteotomy site. The osteotomy was performed with an oscillating saw, with the degree of blade inclination having been determined as part of the preoperative planning. The degree of inclination was based on the decision to lengthen or shorten the metatarsal bone. If the length of the metatarsal bone was to be maintained, the osteotomy was performed perpendicular to the axis of the foot. Otherwise, the osteotomy was performed at an inclination of up to 15°. Care was taken not to violate the lateral cortex with the saw blade, and the final cut was made with an osteotome to preserve the lateral periosteum. The osteotomy was then stabilized with a 2-mm K-wire, which was advanced in retrograde fashion through the incision, close to the bone and along the length of the great toe until it came out from the skin at the distal end of the toe from the medial side, where it was withdrawn until its proximal end reached the osteotomy line. The K-wire was then advanced proximally to the osteotomy level inside the bone until it reached the tarsal bone to which it would be anchored. For this procedure, there is usually no need to resect the medial eminence.

Radiography was performed intraoperatively with a mini C-arm to confirm the displacement and repositioning of the sesamoids under the metatarsal head. The capsule was then closed to help maintain the reduction and further reduce the sesamoids, and this was followed by subcuticular skin closure.

All patients were discharged on the day of the surgery with a backslab and heel post to allow ambulation as tolerated. All patients were followed up in the orthopedic clinic at 2 weeks, 6 weeks, 12 weeks, 6 months, and 1 year after the surgery. At 2 weeks, the backslab was changed to an off-loading shoe. Ambulation was begun at that time. At 6 weeks, the K-wire was removed. An AP radiograph of the foot was taken at the 12-week, 6-month, and 1-year follow-up appointments. The 1-year follow-up examination was the examination upon which the final determination of the status of the reduction and osteotomy, range of motion, and any complication(s) was made. Each radiographic feature and the healing status were assessed by three observers—one involved in the surgery as an assistant and two independent observers not involved in either the surgery or the postoperative care. Differences in opinion were resolved by consensus.

### Data collection and statistical analysis

Clinical and operative data collected for the study included patients’ age, sex, height, weight, comorbidities, smoking history, whether the deformity was of the left foot or right foot, and the time required for HV correction (operation time). Specific HV data were obtained preoperatively and then postoperatively at 1 year and included congruency of the first MTP joint; the HVA, IMA, and DMAA; the Regnauld classification; and position of the sesamoids. Categorical variables are expressed as the number and percentage of patients, and continuous variables are expressed as mean ± standard deviation (SD). Differences between pre- and postoperative categorical variables were analyzed by chi-square test, and differences between pre- and postoperative continuous variables were analyzed by paired *t* test. All statistical analyses were performed with SPSS ver. 23.0 (SPSS Inc., Armonk, NY). A *p* value of < 0.05 was considered statistically significant.

## Results

Characteristics of the 29 study patients are shown in Table 1. Mean age was 34.9 ± 13.6 years (range 20 to 56 years). Ten patients (34.5%) presented with one or more comorbidities, such as asthma, allergy, hypothyroidism, and/or hypertension. Six (20.7%) of the cases were cases of severe HV. Mean operation time was 11.1 ± 2.3 min (range 6.0 to 15.0 min). Four patients (13.8%) reported postoperative pain. No revision surgery was performed in any patient.

**Table 1:** Clinical and operative characteristics of the 29 study patients

Age (years)	34.9 ± 13.6
Height (cm)	158.6 ± 8.8
Weight (kg)	72.9 ± 14.1
Sex	
Male	2 (6.9)
Female	27 (93.1)
Comorbidities	
Asthma	4 (13.8)
Asthma and allergic rhinitis	1 (3.4)
Asthma and skin allergy	1 (3.4)
Hypertension	1 (3.4)
Flat foot	1 (3.4)
Numbness and hypertension	1 (3.4)
Hypothyroidism	1 (3.4)
Smoking history	0
Foot affected	
Right	14 (48.3)
Left	15 (51.7)
Severe hallux valgus	6 (20.7)
Operation time (minutes)	11.1 ± 2.3
Postoperative pain	4 (13.8)
Revision surgery	0

Mean ± SD values or number (%) of patients are shown

The radiographic measurements obtained before and 1 year after the surgery are shown in Table 2. Congruency of the first MTP joint improved significantly from that seen in only 4 cases (13.8%) before surgery to that seen in 17 cases (58.6%) 1 year after surgery (*p* < .001). The mean HVA, IMA, and DMAA were significantly decreased 1 year after surgery (*p* < .001, *p* < .001, and *p* = .023, respectively). The mean decrease in the HVA was − 21.8 ± 7.9° (range − 10 to 44°), and the mean decrease in the IMA was − 7.1 ± 3.5° (range − 14.0 to 0.60°). The IMA increased by 0.60° in 1 patient. The mean decrease in the DMAA was − 7.5 ± 16.9° (range − 60.0 to 20.7°), but the DMAA increased in 9 patients (31.0%).

**Table 2:** Pre- and postoperative radiographic measurements

	Preoperative *n* (%)	Postoperative *n* (%)	*p* value*
Congruency of the 1st MTP joint			
Congruent	4 (13.8%)	17 (58.6%)	< 0.001
Subluxed	25 (86.2%)	12 (41.4%)	
HVA	33.7 (8.3)	12.2 (8.4)	< 0.001
IMA	12.9 (3.2)	5.7 (2.2)	< 0.001
DMAA	29.1 (17.3)	21.5 (1.5)	0.023
Regnauld classification			
1.0	29 (100%)	26 (89.7%)	< 0.001
2.0	–	3 (10.3%)	
Sesamoids			
Subluxed	29 (100%)	13 (44.8%)	< 0.001
Not subluxed	–	16 (55.2%)	

Mean ± SD values or number (%) of patients are shown

*p values < 0.05 were considered significant

Abbreviations: *MTP* metatarsophalangeal, *HVA* hallux valgus angle, *IMA* intermetatarsal angle, *DMAA* distal metatarsal articular angle

**Fig. 1:**
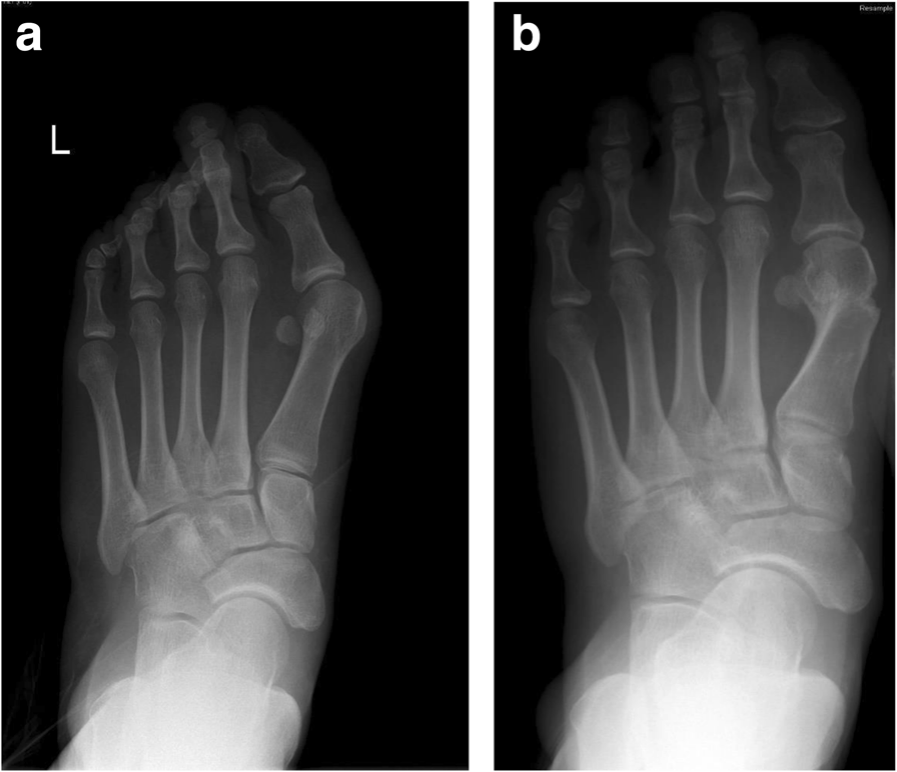
Anteroposterior radiographs of a patient’s left foot obtained before surgical correction of the hallux valgus (**a**, left panel) and 1 year after the surgery (**b**, right panel)

The HVA normalized postoperatively to < 15° in 20 patients (69.0%), the IMA normalized in 25 patients (86.2%), and the DMAA normalized in 4 patients (13.8%). There was also a significant decrease in the number of patients with sesamoid subluxation from 29 (100%) preoperatively to 13 (44.8%) 1 year after the surgery (*p* < .001). However, there were 3 patients (10.3%) in whom the degenerative arthritis of the first MTP joint progressed from Regnauld grade I to Regnauld grade II (*p* < .001). Example radiographs obtained before surgery and 1 year postoperatively are shown in Fig. 1.

## Discussion

Minimally invasive surgical techniques for HV correction have been described extensively in the literature, and they have been judged successful, with the majority of patients reporting a high degree of satisfaction [4, 6]. SERI osteotomy has been used successfully in treating HV without complications, and it has been shown to be cost-effective [7–16]. In this study, we evaluated the efficacy of the SERI technique for HV correction by comparing radiographic measurements obtained preoperatively and 1 year postoperatively, especially to gauge normalization of the HVA, IMA, and DMAA, and we also evaluated the procedure in terms of the development of any postoperative complications.

Overall, the mean HVA, IMA, and DMAA at 1 year after the surgery were significantly decreased. The HVA and the IMA normalized to < 15° and < 9°, respectively, in 69.0% and 86.2% of patients, respectively, whereas the DMAA decreased in only 4 patients (13.8%). Although the HVA, IMA, and DMAA measured radiographically before surgery are known indicators of the severity of HV, the DMAA is the most important for preoperative evaluation of the HV deformity because the DMAA determines the type of operative procedure that will be performed [18]. In comparison to the DMAA, the radiographically measured HVA and IMA measurements have been found to be more reliable in the assessment of HV [24]. Intra- and interobserver reliability for measurement of the DMAA has been poor [25]. With the questionable reliability of the DMAA reflected in our study, assessment of DMAA remains a diagnostic challenge.

Overall, our patients’ radiographically measured HV angles were significantly improved 1 year after the surgery, no complications developed during the 1-year follow-up period, and there was no recurrence. Despite the significant decrease in the HVA, IMA, and DMAA, we found no significant difference in the magnitude of decrease across ages or between age groups. This finding implies that the procedure can be performed safely, regardless of age. Even in cases of severe HV, SERI osteotomy remains an option, as shown by the results of our study. We treated six cases of severe HV, and the outcome was very good in all six cases. Further, there were no postoperative complications in these cases. Our results are in agreement with those of previous studies that showed reliability of the minimally invasive SERI technique (in contrast to traditional techniques) in cases of mild to moderate HV deformity, along with the advantages of substantially shorter operation times and little risk of postoperative complications in [14, 26]. Our study goes a step further in showing reliability of the SERI technique in cases of severe HV. Furthermore, because the SERI osteotomy is a short, day surgery and the only surgical device required is a K-wire, it is cost-effective, and the recovery period is relatively short [27, 28].It is particularly advantageous for young patients, i.e., patients who may not tolerate pain well and may not carefully protect the treated foot/feet until healing is complete [18].

Our study results should be interpreted in light of our study limitations, the first of which is that evaluation of the patients’ HV deformities was based almost solely on the radiographic measurements obtained preoperatively. Second, the study included only 29 patients, and the follow-up data that were analyzed represented only the first year after the surgery. The true measure of a successful outcome of surgical correction of an HV deformity is not only the cosmetic improvement (the decrease in the excessive angulation of the big toe and MTP joint) but also the elimination of clinical signs and symptoms brought about by the HV, including the loss of the great toe’s range of motion and the pain.

## Conclusion

Our study highlighted the safety of the SERI technique, which can be performed even in severe cases with very little risk of postoperative complications. Furthermore, our reliance on radiographic measurements obtained before the surgery and then at 1 year reinforces the value of radiographic assessment and allowed us to document the significant changes that occurred, with no need for further correction and no recurrence, as a result of SERI osteotomy for HV deformities. Further studies in larger series of patients over a longer follow-up period are warranted to confirm the efficacy and advantages of SERI osteotomy performed for HV correction and thus provide a strong rationale for application of the SERI technique throughout the Middle East.

## Abbreviations

AOFAS: American Orthopedic Foot and Ankle Surgery
AP: Anteroposterior
DMAA: Distal metatarsal articular angle
HV: Hallux valgus
HVA: Hallux valgus angle
IMA: Intermetatarsal angle
MTP: Metatarsophalangeal
SERI: Simple, Effective, Rapid, and Inexpensive

## References

[1] Hallux valgus. The American Orthopaedic Foot and Ankle Society. http://www.aofas.org/PRC/conditions/Pages/Conditions/Hallux-Valgus.aspx. Accessed 20 July 2018.

[2] Nix S, Smith M, Vicenzino B (2010). Prevalence of hallux valgus in the general population: a systematic review and meta-analysis. J Foot Ankle Res.

[3] Roddy E, Zhang W, Doherty M (2008). Prevalence and associations of hallux valgus in a primary care population. Arthritis Rheum.

[4] Robinson AH, Limbers JP (2005). Modern concepts in the treatment of hallux valgus. J Bone Joint Surg Br.

[5] Giannini S, Cavallo M, Faldini C, Luciani D, Vannini F (2013). The SERI distal metatarsal osteotomy and scarf osteotomy provide similar correction of hallux valgus. Clin Orthop Relat Res.

[6] Maffulli N, Longo UG, Marinozzi A, Denaro V (2010). Hallux valgus: effectiveness and safety of minimally invasive surgery. A systematic review. Br Med Bull.

[7] Giannini S, Ceccarelli F, Bevoni R, Vannini F (2003). Hallux valgus surgery: the minimally invasive bunion correction (SERI). Tech Foot Ankle Surg.

[8] Bosch P, Wanke S, Legenstein R (2000). Hallux valgus correction by the method of Bosch: a new technique with a seven-to-ten-year follow-up. Foot Ankle Clin.

[9] Maffulli N, Longo UG, Oliva F, Denaro V, Coppola C (2009). Bosch osteotomy and scarf osteotomy for hallux valgus correction. Orthop Clin N Am.

[10] Maffulli N, Oliva F, Coppola C, Miller D (2005). Minimally invasive hallux valgus correction: a technical note and a feasibility study. J Surg Orthop Adv.

[11] Oliva F, Longo UG, Maffulli N. Minimally invasive hallux valgus correction. Orthop Clin North Am. 2009;40:525–30. 10.1016/j.ocl.2009.06.005 19773058

[12] Wu GB, Yang YF, Yu GR, Li B (2014). Comment on Giannini et al.: a minimally invasive technique for surgical treatment of hallux valgus: simple, effective, rapid, inexpensive (SERI). Int Orthop.

[13] Giannini S, Vannini F, Faldini C, Bevoni R, Nanni M, Leonetti D (2007). The minimally invasive hallux valgus correction (SERI). Interact Surg.

[14] Giannini S, Faldini C, Nanni M, Di Martino A, Luciani D, Vannini F (2013). A minimally invasive technique for surgical treatment of hallux valgus: simple, effective, rapid, inexpensive (SERI). Int Orthop.

[15] Wagner E, Ortiz C, Torres K, Contesse I, Vela O, Zanolli D (2016). Cost effectiveness of different techniques in hallux valgus surgery. Foot Ankle Surg.

[16] Trnka HJ, Krenn S, Schuh R (2013). Minimally invasive hallux surgery: a critical review of the evidence. Int Orthop.

[17] Lin YC, Cheng YM, Chang JK, Chen CH, Huang PJ (2009). Minimally invasive distal metatarsal osteotomy for mild-to-moderate hallux valgus deformity. Kaohsiung J Med Sci.

[18] Moscadini S, Moscadini G, Waddell J (2012). Hallux valgus correction in young patients with minimally invasive technique. The role of osteotomy in the correction of congenital and acquired disorders of the skeleton.

[19] Thordarson D, Ebramzadeh E, Moorthy M, Lee J, Rudicel S (2005). Correlation of hallux valgus surgical outcome with AOFAS forefoot score and radiological parameters. Foot Ankle Int.

[20] Baumhauer JF, Nawoczenski DA, DiGiovanni BF, Wilding GE (2006). Reliability and validity of the American Orthopaedic Foot and Ankle Surgery clinical rating scale: a pilot study for the hallux and lesser toes. Foot Ankle Int.

[21] Nery C, Coughlin MJ, Baumfeld D, Ballerini FJ, Kobata S (2013). Hallux valgus in males – part 2: radiographic assessment of surgical treatment. Foot Ankle Int.

[22] Catanese D, Popowitz D, Gladstein AZ (2014). Measuring sesamoid position in hallux valgus. When is the sesamoid axial view necessary?. Foot Ankle Spec.

[23] Shima H, Okuda R, Yasuda T, Jotoku T, Kitano N, Kinoshita M (2009). Radiographic measurements in patients with hallux valgus before and after proximal crescentic osteotomy. J Bone Joint Surg Am.

[24] Coughlin MJ, Freund E (2001). The reliability of angular measurements in hallux valgus deformities. Foot Ankle Int.

[25] Easley ME, Trnka HJ (2007). Current concepts review: hallux valgus part 1: pathomechanics, clinical assessment, and nonoperative management. Foot Ankle Int.

[26] Magnan B, Pezzè L, Rossi N, Bartolozzi P (2005). Percutaneous distal metatarsal osteotomy for correction of hallux valgus. J Bone Joint Surg Am.

[27] Mathavan G, Gaskell L, Pillai A, Thinesh VS, Pravin DC (2015). Minimal invasive hallux valgus surgery: myth or magic. A systematic review. Orthop Rheumatol Open Access J.

[28] Magnan B, Bondi M, Mezzari S, Bonetti I, Samaila E (2013). Minimally invasive surgery of the forefoot: current concept review. Int J Clin Med.

